# Caregiving Challenges During the COVID-19 Pandemic: Findings From the National Poll on Healthy Aging

**DOI:** 10.1093/geroni/igab046.672

**Published:** 2021-12-17

**Authors:** Alicia Carmichael, Natalie Leonard, Jeannette Jackson, Erica Solway, Matthias Kirch, Dianne Singer, Richard Gonzalez, Amanda Leggett

**Affiliations:** 1 University of Michigan, Ann Arbor, Michigan, United States; 2 University of Michigan, Ypsilanti, Michigan, United States

## Abstract

The COVID-19 pandemic posed new challenges for caregivers. This study examines the prevalence of pandemic care challenges (e.g., decreasing care to reduce virus spread, difficulty accessing medical care) and their associations with caregiver mental health and interpersonal well-being in a nationally representative sample of 311 caregivers who participated in the June 2020 National Poll on Healthy Aging. We consider seven care challenges and supports as key predictors of caregiver mental health (care-related stress, self-reported mental health, three depressive symptoms) and interpersonal well-being (lack of companionship, isolation) in bivariate tests and ordinary least squares regressions. Each care challenge/support was endorsed by between 13-23% of caregivers. Difficulty getting needed medical care was the most predictive challenge associated with increased caregiver stress, depressive symptoms, and worsened interpersonal well-being. All care challenges predicted an increase in caregiver stress. Effective caregiver tools and supports must consider changing policies and care needs, especially during a pandemic.

